# Evaluation on Anti-Inflammatory, Analgesic, Antitumor, and Antioxidant Potential of Total Saponins from *Nigella glandulifera* Seeds

**DOI:** 10.1155/2013/827230

**Published:** 2013-02-20

**Authors:** Jun Zhao, Fang Xu, Hua Huang, Zhengyi Gu, Linlin Wang, Wei Tan, Jinhua He, Yan Chen, Chenyang Li

**Affiliations:** Xinjiang Key Laboratory for Uighur Medicine, Institute of Materia Medica of Xinjiang, Urumqi 830004, China

## Abstract

*Nigella glandulifera* seeds are used as a spice or remedy for the treatment of various inflammatory diseases. This study aimed to investigate analgesic (writhing test), anti-inflammatory (ear-induced edema, vascular permeability test), antioxidant, and antitumor activities of total saponins from this plant (TSN). TSN (6, 12, and 24 mg/kg) were exhibited analgesic and anti-inflammatory activities in a dose-dependent manner (*P* < 0.05). In D-galactose-induced ageing model, TSN significantly increased the plasma superoxide dismutase (SOD) and glutathione peroxidase (GSH-Px) activities (*P* < 0.05) and decreased the malondialdehyde (MDA) level compared to control group (*P* < 0.05). DPPH radical scavenging effect of TSN was also found. Moreover, TSN (20 mg/mL) showed 86.75% and 88.26% inhibition of the growth on Bel-7402 and Hela cells, respectively. Five compounds were further isolated and identified from TSN as Nigella A, B, C, D, and nigeglanoside, of which the content of Nigella A was 60.36 ± 1.25 g/100 g TSN by HPLC-ELSD method. Altogether, these results suggest that TSN could be considered as a potential analgesic, anti-inflammatory, antitumor, and antioxidant agent.

## 1. Introduction

Nigella (*Nigella sativa* L., *Nigella glandulifera *Freyn et Sint, and *Nigella damascena,* L. etc.), belonging to the buttercup family Ranunculaceae, is commonly known as black cumin (black seeds) [1]. Nigella seeds are widely used for medicinal purposes as a natural remedy for a number of illnesses such as hypertension, diabetes, inflammation, bronchitis, headache, and gastrointestinal disturbances [2]. In recent years, voluminous research has been carried out on the medicinal properties of the seeds as antioxidant, antimicrobial, anti-inflammatory, and anticancer agents [3–5]. These properties have been attributed to a variety of active constituents in seeds and its fixed oil [6, 7], of which saponins are mainly characteristic compounds of water-soluble extracts from Nigella [8]. Pharmacological effects of saponins have been reported in many references, and these compounds are considered to be beneficial to the health of mankind [9, 10]. 

*N. glandulifera *Freyn is widely distributed in Xinjiang, Yunnan, and Tibet of China and is now mainly cultivated in the Taklimakan Desert edge region in Xinjiang [11]. The seeds of *N. glandulifera *are frequently added to “naan” (a kind of crusty pancake, favorite food of the Uygur and Kazak people) as a spice, and its water decoction is used in Uighur's traditional medicine for the treatment of numerous disorders such as diuretic, analgesic, insomnia, dizziness, tinnitus, amnesia, and bronchial asthma [12, 13]. Several classes of compounds have been isolated from water-soluble extracts of *N. glandulifera*, such as alkaloids, flavonoids, and saponins [14, 15]. Saponins with the highest content (64.5%) are mainly characteristic compound of *N. glandulifera* (TSN). However, the biological activities of saponins from this plant were rarely reported so far. Therefore, the aim of this study was to investigate anti-inflammatory, analgesic, anti-tumor, and antioxidant potential of total saponins from *N. glandulifera *by experiments *in vivo *or *in vitro*. 

## 2. Material and Methods

### 2.1. Chemicals

2,2-Diphenyl-1-(2,4,6-trinitrophenyl)hydrazyl (DPPH) and dimethyl sulfoxide (DMSO) were purchased from Sigma-Aldrich (St. Louis, MO, USA). Superoxide dismutase (SOD), glutathione peroxides (GSH-Pxs), and malondialdehyde (MDA) assay kits were supplied by Nanjing Jiancheng Bioengineering Institute (Nanjing, China). RPMI 1640 medium was obtained from Gibco Co. (USA). HPLC-grade acetonitrile was purchased from Fisher BioReagents (NJ, USA). Yemugua tablet (YMGP) was purchased from Hepin Pharmacy Co. (Guangdong, China). The other chemicals and solvents used in this experiment were of the highest quality available.

### 2.2. Plant Material

*N. glandulifera* were collected from Aksu, Xinjiang, in China, in July 2011. The plant material was identified by the associate researcher Jiang He, Xinjiang Institute of Material Medica. A voucher specimen was deposited at Xinjiang Institute of Material Medica in China.

### 2.3. Preparation of TSN

The powdered seeds (10.0 kg) were defatted at reflux condition with petroleum ether and extracted with 30% ethanol by exhaustive maceration to yield a dark brown residue (2.2 kg). After being dissolved in water, the extract was purified by AB-8 adsorption macroporous resin to obtain total saponin extracts from *N. glandulifera* (TSN, 210 g). TSN were applied to ODS RP-18 column and eluted with mixtures of MeOH : H_2_O (0 : 1 → 1 : 0) successively. Elutes were combined into five subfractions according to TLC behavior using two solvent systems CHCl_3_ : MeOH : H_2_O (6 : 4 : 0.5) and BuOH : AcOH : H_2_O (4 : 1 : 1) (spots were visualized after spraying 10% H_2_SO_4_). Various fractions were repeatedly purified by Sephadex LH-20 column with methanol, and five saponins were isolated from TSN, and their structures were confirmed using MS, ^1^H, and ^13^C NMR (References). The purity of the saponins was determined to be more than 95% compared with the peak areas detected by HPLC-ELSD.

### 2.4. HPLC-ELSD Analysis of TSN

The high-performance liquid chromatography (HPLC) (LC-10A HPLC instrument, Shimadzu Co., Japan) was employed to analyze the percentage contents of Nigella A in TNS. A Cosmosil-C18 column (250 mm × 4.6 mm, 5 *μ*m) and a Zorbax ODS C18 guard column (12.5 mm × 4.6 mm, 5 *μ*m) were used at 40°C. A binary gradient elution system consisted of water (A) and acetonitrile (B) and separation was achieved using the following gradient program: 0–15 min, 20–40%B; 15–20 min, 40%B; 20–22 min, 40–20%B; and 22–25 min, 20%B. The flow rate was at 1.0 mL/min and the sample injection volume was 5 *μ*L. The all tech ELSD impactor was set at ON mode, the drift tube temperature was 65°C, and the nebulizer nitrogen gas flow rate was at 2.0 L/min.

### 2.5. Animals

The study was conducted on Kunming mice weighting 18 ± 22 g (The Experimental Animal Center in Xinjiang, China; SCXK (Xin) 2003–2001). Animals were kept under a 12 h/12 h light/dark cycle and allowed free access to food and water. The study protocols were approved by the Ethics Committee on Animal Experiment, Xinjiang Material Medica, China.

### 2.6. Cells

The human hepatoma carcinoma cell lines Bel 7402 and cervical carcinoma cell lines Hela were provided by the Institute of Materia Medica, Chinese Academy of Medical Sciences, and maintained with RPMI 1640 medium containing 10% fetal bovine serum and 100 ng/mL, each, of penicillin and streptomycin at 37°C in a humidified atmosphere with 5% CO_2_.

### 2.7. Acute Oral Toxicity Study in Mice

TSN were subjected to acute oral toxicity studies as per SFDA guidelines no. [Z] GPT2-1 [16]. Mice were randomly assigned to each of three groups of 30 mice (15 females and 15 males). They were fasted overnight (12 h) with free access to water prior to administration of doses (48 and 24 g/kg) of TSN dissolved in distilled water. The control group mice were p.o. administered with distilled water. The animals were observed continuously for first 72 hours and 14 days for any signs of mortality, body weight, toxicity, behavioral, and viscera changes. On day 15, mice were killed and all organs and tissues were observed macroscopically. 

### 2.8. Cytotoxicity Assays

The human hepatoma carcinoma cell lines Bel-7402 and cervical carcinoma cell lines Hela were cultured in RPMI 1640 medium supplemented with 10% fetal bovine serum (FBS), 100 U/mL penicillin, and 100 *μ*g/mL streptomycin, at 37°C in an incubator containing 5% CO_2_. Cells were passaged every 2days using Trypsin (0.25%) solution. Exponentially growing cells were used for experimentation. Cell viability assay was performed with MTT photometric analysis, as first described by Mosmann [17], with slight modification. Briefly, 4.5 × 10^5^/mL was seeded in 96-well microtiter plates at 200 *μ*L/well. Twenty-four hours after treatment, the supernatant was removed. TNS were made into 20, 10, 5, 2.5, 1.25, 0.625, 0.3125, and 0.156 mg/mL in cultured solution and added to 96-well microtiter plates. Fourty-eight hours after treatment, 20 *μ*L MTT reagent (5 mg/mL) was added. After 4 h incubation at 37°C, 200 *μ*L of DMSO was added and plates were oscillated for 10 min in a balance oscillator. The extent of the MTT reduction was measured by a plate reader at a wavelength of 570 nm. The inhibitory rate of cell proliferation was calculated as follows: inhibitory rate (%) = (1 − experimental group A value/control group A value) × 100%.

### 2.9. Determination of Antioxidant Activity by DPPH Radical Scavenging Ability

The effects of TSN on DPPH radicals were estimated according to the method of Zhao et al. [18]. Aliquots of TSN at various concentrations were mixed with 2.0 vols of 6.5 × 10^−5 ^M solution of DPPH. The mixture was shaken vigorously and left to stand at room temperature for 30 min in the dark. The absorbance of the reaction solution was measured spectrophotometrically at 517 nm. The percentages of DPPH decolorization of the samples were calculated according to the equation: radical scavenging activity (%) = [1 − (ABS sample/ABS control)] × 100. EC_50_ value was defined as the concentration (in mg/mL) of the extract required to deplete the amount of DPPH radical by 50%. BHT was used as a positive control. 

### 2.10. Antioxidant Effect of TSN in Ageing Mice

After 1 week of acclimatization, the mice were randomly divided into five groups (12 mice per group) and i.p. injected with 0.1 mL/10 g of 5% D-galactose once daily for 6 weeks except normal control group (i.p injected with 0.3 mL of physiological saline) [19]. Simultaneously, normal control group mice and ageing model group mice were p.o. administered with 0.1 mL/10 g of 20% Arabic gum each, respectively; TSN treatment group mice were p.o. administered with a different dose of extract (8.8, 17.5, and 35 mg/kg). Following the 35-day treatment process and 24 h after the last administration, all 50 mice were weighed then sacrificed by the humane method of cervical dislocation. The liver was surgically excised from the animal, accurately weighed, and then homogenized immediately in ice-cold 0.9% NaCl solution (0.1 g tissue/mL solution). The suspension was centrifuged at 4000 rpm/min at 4°C for 10 min, and the supernatant was collected for further analysis. The activities of SOD, GSH-Px, and the level of MDA were measured using commercially available kits in accordance with instructions. 

### 2.11. Analgesic Activities

The analgesic activity of TSN was investigated using acetic acid-induced writhing response in mice, and the test was carried out using the method of Muhammad et al. [20]. This method was used to preferentially evaluate possible peripheral effects of TSN as analgesic substance. Five groups of Kunming male mice (*n* = 10) were fasted overnight prior the start of the experiment, and water ad libitum. The peripheral analgesic drug, YMGP, was used as a positive control. Group 1 received the vehicle-distilled water (10 mL/kg, p.o.), and group 2 was treated with YMGP (928 mg/kg, p.o.), whereas groups 3, 4, and 5 animals were orally administered with TSN at doses of 6, 12, and 24 mg/kg. Sixty minutes after treatment, the mice were injected (i.p.) with 0.1 mL/10 g body of 0.7% acetic acid solution to induce the characteristic writhings. After 5 min, the mice were placed in an observation box, and the number of writhes in a 15 min period was counted. Antinociception (analgesia) is expressed as the reduction of the number of writhing movements between control animals (acetic-acid-treated mice) and mice pretreated with these compounds and then acetic acid.

### 2.12. Anti-Inflammatory Activity

The anti-inflammatory activity of TSN was investigated using the following models.

#### 2.12.1. Xylene-Induced Ear Edema in Mice

Antiacute inflammatory activity was determined by xylene-induced mice ear edema [21]. Fifty mice were equally divided into five groups randomly including control group (distilled water), YMGP-positive control group (928 mg/kg body wt), and TSN groups (6, 12, or 24 mg/kg body wt). The vehicle and drugs were administered orally, respectively, once per day for 3 days. One and half hour after the last administration of drugs, inflammatory response was induced on the inner and external surface of the right ear (surface: about 1 cm^2^) by application of 20 *μ*L xylene. 30 min later, mice were sacrificed by cervical dislocation and a section (Ø 6 mm) of ears was removed from both the treated (right) and the untreated (left) ears. Edema rate was measured as the percentage of the weight difference between the two ear discs compared to the untreated (left) ears. The anti-inflammatory activity was expressed as the percentage of inhibition in treated mice compared to the control mice. 

#### 2.12.2. Vascular Permeability Test

The method of Koo et al. [22] was used to evaluate the effect of the extract on vascular permeability in adult albino mice of both sexes. Fifty mice were equally divided into five groups randomly including control group (distilled water), YMGP-positive control group (928 mg/kg body wt), and TSN groups (6, 12, or 24 mg/kg body wt). One and half hour after oral administration of TSN and YMGP, 0.2 mL of Evans Blue dye (0.5% in Normal saline) was intravenously administered through the tail vein. Subsequently, animals received intraperitoneal injection of 1.0 mL/100 g of acetic acid (0.6%, v/v). Treated animals were sacrificed 20 min after acetic acid injection and the peritoneal cavity washed with normal saline (6.0 mL) into heparinized tubes and centrifuged 15 min at 3000 rpm. The dye content in the supernatant was measured at 590 nm using spectrophotometer.

### 2.13. Statistical Analysis

The data obtained were computed using SPSS 11.5 software and later analyzed using ANOVA of variance. The Duncan test with significance level of 0.05 between means was used.

## 3. Results and Discussion

### 3.1. Phytochemical Study

**Figure 1:**
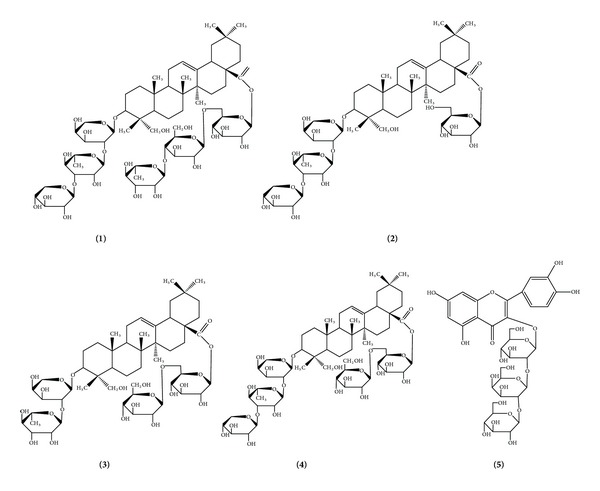
Chemical structure of compounds.

**Figure 2:**
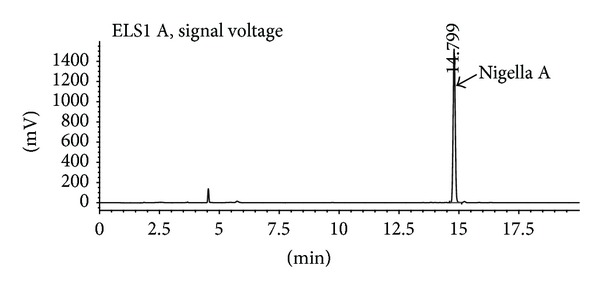
HPLC chromatogram of TSN solution.

Five compounds were isolated from TSN and elucidated as 3-*O*-[D-xylopyranosyl-(1 → 3)-*α*-L-rhamnopyranosyl-(1 → 2)-*α*-L-arabinpyranosyl]-28-*O*-[-*α*-L-rhamnopyranosyl-(1 → 4)-*β*-D-glucopyranosyl-(1 → 6)-*β*-D-glucopyranosyl] hederagenin (Nigella A, **1**), 3-*O*-[D-xylopyranosyl-(1 → 3)-*α*-L-rhamnopyranosyl-(1 → 2)-*α*-L-arabinpyranosyl]-28-*O*-*β*-D-glucopyranosyl hederagenin (Nigella B, **2**), 3-*O*-[*α*-L-rhamnopyranosyl-(1 → 2)-*α*-L-arabinpyranosyl]-28-*O*-[*β*-D-glucopyranosyl-(1 → 6)-*β*-D-glucopyranosyl] hederagenin (Nigella C, **3**), 3-*O*-[D-xylopyranosyl-(1 → 3)-*α*-L-rhamnopyranosyl-(1 → 2)-*α*-L-arabinpyranosyl]-28-*O*-[-*α*-L-rhamnopyranosyl-(1 → 4)-*β*-D-glucopyranosyl-(1 → 6)-*β*-D-glucopyranosyl] hederagenin (Nigella D, **4**), and nigeglanoside (**5**) by extensive spectroscopic methods including 1D-(^1^H, ^13^C) NMR experiments (Figure 1). All data were compared with those in previous literature [23–25]. Among these compounds, the content of Nigella A from TNS was far above others. Therefore, the content of Nigella A was determined by HPLC-ELSD, and its content is 60.36 ± 2.5 g/100 g in TSN (Figure 2).

### 3.2. Acute Toxicity

Following administration of TSN, the mice were monitored daily for mortality, clinical signs, and gross changes in appearance and behavior. No deaths occurred in the test and control group. There were no significant differences in body weights between the TSN treated and control group in either sex (data not reported in this paper). The result indicated that treatment of TSN was safe under the maximum dose at 48 g TSN/kg body. 

### 3.3. Cytotoxicity Activity

**Figure 3:**
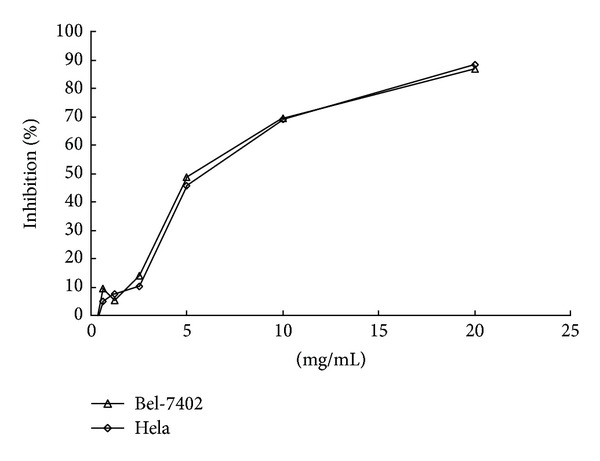
Inhibiting effect of TSN on human hepatoma carcinoma cell lines Bel 7402 and cervical carcinoma cell lines Hela growth. Results are mean ± SD (*n* = 4).

Two cell lines, that is, Bel-7402, and Hela cells, were used to test the effect of TSN on their cell proliferation. TSN showed significant inhibitory effects on these two cell lines, which were in a dose-dependant manner. TSN at the dose of 20 mg/mL showed 86.75% and 88.26% inhibition of the growth of Bel-7402 and Hela cells, respectively (Figure 3).

### 3.4. DPPH Radical Scavenging Activity

**Figure 4:**
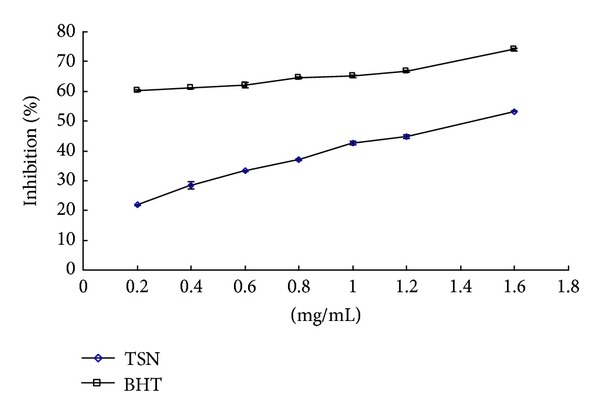
DPPH radical scavenging activity of TSN. Results are mean ± SD (*n* = 3).

The scavenging capability of DPPH radical was determined by the decrease in its absorbance at 517 nm under effect of antioxidants. Due to rapid hydrogen-accepting ability, DPPH reacted with antioxidants and converted into 1,1-diphenyl-2-picrylhydrazin, shows decrease in absorbance simultaneously [26]. The degree of discoloration indicates the scavenging potential of the antioxidant extracts [27].  Figure 4 showed the DPPH free radical scavenging activities of TSN. DPPH was reduced by TSN in a concentration-dependent manner.  Figure 4 shows the dose response curves of DPPH radical scavenging activities of TSN, at a concentration range of 0.2–1.6 mg/mL, and percent inhibition and the IC_50_ of TSN were 21.87–53.28% and 1.58 mg/mL, respectively. BHT as a reference drug scavenged the DPPH radical by inhibitions of 60.31–74.01% and the IC_50_ value of 58.07 *μ*g/mL.

### 3.5. The Effect of TSN on the Activities of SOD, GSH-Px, and MDA in the Liver of Aged Mice

Ageing is a natural process in all living organisms, and oxidative stress is one of root causes of the ageing process [28]. The importance of antioxidant enzymes is generally emphasized in the prevention of oxidative stresses by scavenging of ROS [29, 30]. The antioxidant system comprises several enzymes such as SOD and GSH-Px [31–33]. The effect of TSN on the activities of SOD, GSH-Px, and the levels of MDA in the liver of aged mice is shown in Table 1. A marked increase in MDA and significant decrease (*P* < 0.05) of antioxidant enzymes activity (SOD, GSH-Px) were observed in the liver between the treatments of normal control group and model control group. TSN treatment significantly inhibited (*P* < 0.05) the formation of MDA in the liver and raised the activity of antioxidant enzymes in a dose-dependent manner. The administration of TSN to the D-galactose-treated mice with 8.8, 17.5, and 35 mg/kg increased both SOD and GSH-Px enzymatic antioxidant liver activity (*P* < 0.01 and *P* < 0.05).

### 3.6. Anti-Inflammatory and Analgesic Activities

The peripheral analgesic effect may be mediated through inhibition of cyclo-oxygenases and/or lipoxygenases (and other inflammatory mediators), while the central analgesic action may be mediated through inhibition of central pain receptors [34]. The acetic-acid-induced writhes have often been used to evaluate the peripherally and centrally acting analgesic drugs [35]. YMGP is the most popular medication for rheumatism arthritis pain, sciatica, and prosopalgia in Chinese medicine. Therefore, we select YMGP as a reference drug. 

Peripheral analgesic activity was assessed by acetic-acid-induced writhing test, which showed significant (*P* < 0.01 and *P* < 0.05) suppression of writhes (Table 2). Acetic acid is known to trigger the production of noxious substances within the peritoneum, which induces the writhing response. The effect of TSN against the noxious stimulus may be an indication that it depressed the production of irritants and thereby bringing a reduction in the number of writhes in animals. Injection of acetic acid into the control mice resulted in 71 ± 19 writhes. Pretreatment with TSN at the doses of 6, 12, and 24 mg/kg reduced the number of writhes to 38 ± 8 (46.5% inhibition), 33 ± 9 (53.6% inhibition), and 29 ± 8 (59.2% inhibition) at a dose-activity dependence relationship, respectively. Interestingly, the analgesic activity of high dose was higher than that of YMGP (33 ± 8 writhes, 54.5% inhibition). It was also observed that the onset of writhing was delayed and the duration of writhing was shortened with TSN pretreatment.

**Figure 5:**
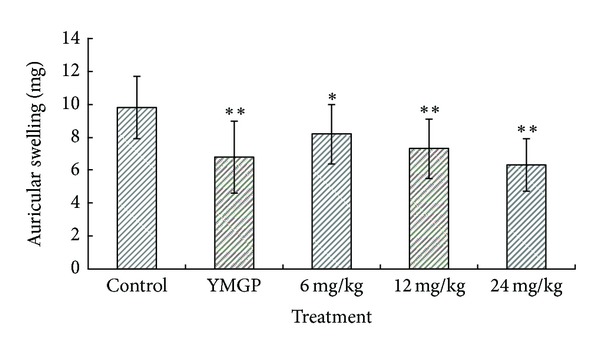
Effects of TSN and YMGP on xylene-induced ear oedema in mice. Values are mean ± SD of differences in weight between right and left ears of animals. *n* = 10. Control (vehicle): distilled water; YMGP: Yemugua tablet; TSN: total saponins from *Nigella glandulifera*.*P < 0.05 and **P < 0.01, compared with corresponding control.

Xylene-induced mouse ear edema reflects the oedematization during the early stages of acute inflammation, which was probably related to the release and inhibition of the inflammation factors [36]. In xylene-induced ear edema, the oral administration of TSN suppressed significantly xylene-induced ear oedema in mice. The oedema inhibitory rates of casticin were 16.6%, 25.9%, and 35.5% at doses of 6, 12 and 24 mg/kg, respectively, whereas YMGP (928 mg/kg) produced 30.53% inhibitory rate compared with control (Figure 5). 

**Figure 6:**
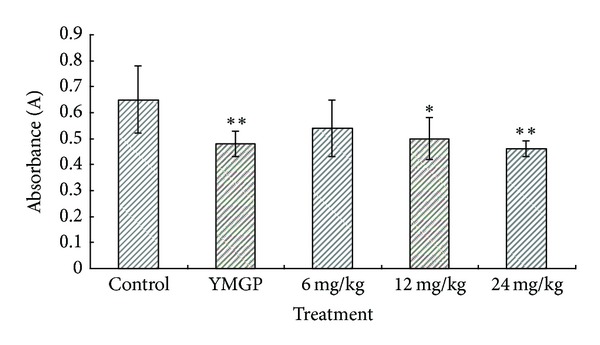
Effects of TSN and YMGP on acetic-acid-induced increased vascular permeability in mice. Values are mean ± SD, *n* = 10. Control (vehicle): distilled water; YMGP: Yemugua tablet; TSN: total saponins from *Nigella glandulifera*. *P < 0.05 and **P < 0.01, compared with corresponding control.

The inflammatory response is a physiological characteristic of vascularized tissues also [37]. Exudation, which is a consequence of increased vascular permeability, is considered a major feature of acute inflammation. In the inflammatory reaction, increased vascular permeability leads to exudation of fluid rich in plasma proteins including immunoglobulins, coagulation factors, and cells into the injured tissues [38] (with subsequent edema at the site). Therefore, inhibition of increased vascular permeability can modulate the extent and magnitude of the inflammatory reaction [39, 40]. Chemical-induced vascular permeability causes an immediate sustained reaction that is prolonged over 24 h, and its inhibition suggests that the extract may effectively suppress the exudative phase of acute inflammation [41], and acetic-acid-induced increased vascular permeability in mouse model is a typical capillary permeability assay [42, 43]. In this study, TSN significantly reduced the increased peritoneal vascular permeability, indicating the suppression of the vascular response in the process of acute inflammation (Figure 6). Oral administration of 6, 12, and 24 mg/kg of TSN evoked a significant (*P* < 0.05) dose-related inhibition of vascular permeability induced by acetic acid in mice. At the doses of 6, 12, and 24 mg/kg, TNS produced 17.9%, 23.9%, and 29.4% inhibition of dye leakage, respectively, with dose-depended manner, while YMGP produced 26.9% inhibition.

## 4. Conclusion

In summary, our results suggest that TSN possesses anti-inflammatory, analgesic, antitumor, and free radical scavenging activities and provide some scientific evidence to support the folk medicinal utilization of *N. glandulifera*. Furthermore, TSN is a good candidate for the development of new anti-inflammatory and analgesic medicine compared with efficacy of YMGP. Therefore, TSN may be worth further investigating and elucidating. 

## Figures and Tables

**Table 1:** Effect of TSN on MDA, SOD, and GSH-Px in liver in D-galactose-induced ageing mice.

Group	Dose (mg/kg·d)	MDA (nmol/mgprot)	SOD (U/mgprot)	GSH-Px (U/mgprot)
Control	—	1.181 ± 0.187	259.785 ± 34.007	577.273 ± 95.582
Model	—	1.789 ± 0.383^##^	173.843 ± 21.939^##^	270.775 ± 35.582^##^
	8.8	1.296 ± 0.206**	181.220 ± 13.194	300.344 ± 26.338**
TSN	17.5	1.252 ± 0.398**	194.609 ± 18.456*	354.958 ± 69.549**
	35	1.236 ± 0.315**	203.477 ± 17.719**	411.949 ± 57.553**

Values are expressed as mean ± SD (*n* = 12).

^##^ *P* < 0.01, compared with control group; *P < 0.05, **P < 0.01, compared with model group.

**Table 2:** Effects of TSN on acetic-acid-induced writhing response in mice.

Treatment group	Dose (mg/kg)	Number of writhes	Inhibition (%)
Control	—	71 ± 19	—
YMGP	928	33 ± 8**	54.5
	6	38 ± 8**	46.5
TSN	12	33 ± 9*	53.6
	24	29 ± 8**	59.2

Values are expressed as mean ± SD (*n* = 10).

Control (vehicle): distilled water; YMGP: Yemugua tablet; TSN: total saponins from *Nigella glandulifera*. **P < 0.01, *P < 0.05, compared with corresponding control.
